# Supplementary material for: Magnetically Modified Biosorbent for Rapid Beryllium Elimination from the Aqueous Environment
Source: Materials (Basel). 2021 Nov 3;14(21):6610. doi: 10.3390/ma14216610 (PMC8585364; doi:10.3390/ma14216610)
Supplement: Supplementary file 1 [file materials-14-06610-s001.zip › materials-1365999-supplementary.pdf]

materials

MDPI

Supplementary

Magnetically Modified Biosorbent for Rapid Beryllium Elimination from the Aqueous Environment

Michaela Tokarčíková *, Oldřich Motyka, Pavlína Peikertová, Roman Gabor and Jana Seidlerová

Nanotechnology Centre, Energy and Environmental Technology Centre, VŠB-Technical University of Ostrava, 708 33 Ostrava-Poruba, Czech Republic; oldrich.motyka@vsb.cz (O.M.); pavlina.peikertova@vsb.cz (P.P.); roman.gabor@vsb.cz (R.G.); jana.seidlerova@vsb.cz (J.S.)
* Correspondence: Michaela.tokarcikova@vsb.cz; Tel.: +420-597-321-549

The non-linear form of the Freundlich [1] sorption isotherm model can be expressed as:

$$q_e = K_F \times c_e^{1/n} \tag{S1}$$

where K_F (mg/g) corresponds to the Freundlich constant and $1/n$ refers to the heterogeneity of the adsorbate sites [2].

The Langmuir sorption isotherm [3] in non-linear form is expressed as follows:

$$q_e = \frac{q_m \times K_L \times c_e}{1 + K_L \times c_q} \tag{S2}$$

K_L (L/mg) is the Langmuir constant related to the energy of sorption and reflects the affinity of the resin towards the metal ions [2].

The separation factor R_L correlated to the values of initial concentration C_i and the Langmuir constant K_L and determined whether a sorption system is favorable or unfavorable [4]. R_L value was calculated at maximum beryllium concentration as follows:

$$R_L = \frac{1}{1 + K_L \times C_i} \tag{S3}$$

$R_L = 0$ characterizes irreversible sorption, $0 < R_L < 1$ signifies favorable sorption, $R_L = 1$ shows linear sorption and $R_L > 1$ indicates unfavorable sorption [5].

The Sips sorption isotherm combines the Langmuir and the Freundlich sorption isotherm models. The Sips isotherm is used for predicting sorption in a heterogeneous system.

$$q_e = \frac{q_m \times K_S \times c_e^{\frac{1}{n_s}}}{1 + K_s \times c_e^{\frac{1}{n_s}}} \tag{S4}$$

K_S (L/g) and n_s (L/mg) are the Sips isotherm models constants representing the surface heterogeneity [6]. At low adsorbate concentrations, it predicts the Freundlich isotherm model, while at higher adsorbate concentrations, it predicts the characteristic monolayer sorption of the Langmuir model [7,8].

The Redlich-Peterson sorption isotherm is described:

$$q_e = \frac{A \times c_e}{1 + B \times c_e^g} \tag{S5}$$

where A and B are the Redlich-Peterson constants, exponent g reflects the heterogeneity of the sorbent, which lies between 0 and 1 [9].

References

1. Freundlich, H.M. Over the Adsorption in Solution. *J. Phys. Chem. A*. **1906**, *57*, 385–470.
2. Taha, M.H. Sorption of U(VI), Mn (II), Cu(II), Zn(II), and Cd(II) from multi-component phosphoric acid solutions using MARATHON C resin. *Environ. Sci. Pollut. Res.* **2021**, *28*, 12475–12489, doi:10.1007/s11356-020-11256-3.
3. Langmuir, I. The adsorption of gases on plane surfaces of glass, mica and platinum. *J. Am. Chem. Soc.* **1918**, *40(9)*, 1361–1403, doi:10.1021/ja02242a004.
4. McKay, G.; Blair, H.S.; Gardner, I.R. Adsorption of dyes on chitin. I. Equilibrium studies. *J. Appl. Polym. Sci*. **1982**, *27*, 3043–3057, doi:10.1002/app.1982.070270827.
5. Eren, Z.; Acar, F.N. Adsorption of Reactive Black 5 from an aqueous solution: Equilibrium and kinetic studies. *Desalination*. **2006**, *194*, 1–10, doi:10.1016/j.desal.2005.10.022.
6. Sips, R. On the Structure of a Catalyst Surface. II. *J. Chem. Phys*. **1950**, *18*, 1024–1026, doi:10.1063/1.1747848.
7. Vijayaraghavan, K.; Padmesh, T.V.N.; Palanivelu, K.; Velan, M. Biosorption of nickel(II) ions onto Sargassum wightii: Application of two-parameter and three-parameter isotherm models. *J. Hazard. Mater*. **2006**, *133*, 304–308, doi:10.1016/j.jhazmat.2005.10.016.
8. Tanhaei, B.; Ayati, A.; Iakovleva, E.; Sillanpää, M. Efficient carbon interlayed magnetic chitosan adsorbent for anionic dye removal: Synthesis, characterization and adsorption study. *Int. J. Biol. Macromol*. **2020**, *164*, 3621–3631, doi:10.1016/j.ijbiomac.2020.08.207.
9. Redlich, O.; Peterson, D.L. A useful adsorption isotherm. *J. Phys. Chem*. **1959**, *63*, 1024, doi:10.1021/j150576a611.
